# The Third-Class Carriage

**Published:** 1890-01-25

**Authors:** 


### The Third-Class Carriage.

I often like to travel third-class in a railway carriage, because the type of individual one rubs shoulders with affords to the professional eye interesting observation and study. There are many persons who disdain taking a third-class ticket because they may come into contact with the unwashed; because of the babies which are there; because the carriages are not comfortable; but chiefly because they consider it *infra dig.* So people who can ill-afford it frequently take the more expensive tickets, having nothing to show for the additional outlay when they arrive at the journey's end. I recognise some of these sort of people at a glance. A type is the *silver'd* youth—not the real gilded youth—who journeys at the cost of his father the clerk, or of his mother the widow. The youth who knows not of the sage Goethe, who said "No youth can be a master," and who thinks himself the centre of the universe. Another type is the female who "has seen better days," who now probably invites boarders, not lodgers, to her domicile, but who nevertheless fears to meet her friends, and lives in perpetual dread of Mrs. Grundy. A third type is the gentleman—maybe a compulsorily retired officer—whose whilom fashionable garments bear evidence of the most careful brushing, whose hat is too glossy to be new, and whose boots are scarcely in that pristine condition which might be desired. Let us, however, revert to the *bonâ fide* third-class passengers. Opposite is sitting a good-looking, neatly-dressed young woman, with a baby in arms, and attended by a boy about three years old—evidently the family of some well-to-do mechanic or small shopkeeper. Her errand is patent to the medical eye. One of the children has enlarged glands of the neck, the other has a knee covered with bandages. Evidently the ailments are scrofulous, and they have been to some hospital. The first seems a fit subject for Mr. Treeve's operation; the second appears to require that perfect rest of the limb only secured by mechanical restraint. But where did these children acquire their scrofulous taint from? Not from the mother apparently, who looks anything but scrofulous. A vision of perhaps a consumptive father arises. But we are justified in hoping not, because, like all hereditary diseases, the scrofulous diathesis may avoid one generation to reappear in another. But sitting by our side is a man apparently half asleep. It does not take long to diagnose what is the matter with him! He has taken rather too much to drink, but fortunately he is not in the argumentative or quarrelsome stage of inebriety. His bloated face, and especially his jolly nose—not "hammer nose," as explained by Dr. Balmanno Squire—evidences that with him excess is habitual. As a very general rule these sort of persons do not leave off drinking, and they will be less likely to do so than ever, if they hear of the statistics recently published by the committee of the British Medical Association appointed to enquire into the longevity of teetotallers, moderate and excessive drinkers. The committee say that teetotallers are a comparatively short-lived race, and that although drunkards often come to grief, moderate drinkers have on the whole the best chance. Nevertheless we know that alcohol does produce injurious effects. Will our friend in the railway carriage suffer from cirrhosis of the liver, or will he be the subject of renal disease, or will his arteries become diseased? If we knew the kind of liquor he affects a prophecy might be made. But it would scarcely do to appear inquisitive to a drunken man!

Next we have the scarlet coat of a volunteer, the wearer being a hale and hearty fresh-looking young man. Judging from his hands we should say he did not get his living by manual labour. Perhaps a clerk in an office, or a shopman, or more probable, considering his fresh and healthy appearance, a person employed outdoor. We should say he is perfectly healthy and strong, and one who would delight the heart of a recruiting sergeant if he took the shilling. And this leads to the reflection why a great many more of our young men do not take the shilling. Service in the ranks ought to hold its own in competition against most kinds of employment, but for some reason or other it does not do so. We cannot suppose it is because there is a remote possibility of the soldier being shot at. Britons, as a rule, never were cowards, are not cowards now, and never will be cowards. A soldier has enough to eat, often by some means or other more than enough to drink, he is, as a rule, well housed, his work is not so arduous as that of a policeman, he has an opportunity of seeing foreign countries, and on leaving Her Majesty's service, if with good character, he seldom has difficulty in obtaining employment, which, with the pension he receives, enables him to live fairly well. There are also opportunities afforded in the army for good men to rise to the non-commissioned ranks, and occasionally a man commencing as a private soldier ends as a commissioned officer. Notwithstanding all this, men do not flock to the army. For this we believe there are two main reasons: First, although a man will submit to occasional discipline as a volunteer, he dislikes placing himself in a position where he has to submit to perpetual discipline—where practically he is no longer his own master. Secondly, because there is still a relic of the old feeling left, which regarded the soldier as a lost man. The phrase, "gone for a soldier," so frequently used by the classes from which soldiers are recruited, expresses this feeling forcibly. But the number of men now seen about the streets of London wearing military medals, or otherwise affording evidence of having been soldiers, but now in some other employment, should be sufficient contradiction to the impression that when a man becomes a soldier he is "gone." While thus reflecting we stop at a small station, and a family, evidently from the country, enter the carriage. *Paterfamilias* looks very like a farmer in a small way; and *materfamilias*, a portly dame, fat, good-looking, if not exactly fair, and at least forty, appears as if she were well able to manage the household, butter, eggs, chickens, etc., of a farm of any size whatever. The olive branches are five in number, only one of whom attracts attention. The child, a boy of about four years of age, has paraplegia, not happily complete, and without enquiry we may surmise this condition followed some acute disease—possibly diphtheria. After the manner of country people of the class coming up to London for the day, no sooner do they enter the carriage than a basket, carried by the eldest daughter, is opened, and refreshments in the shape of, apparently, sausage sandwiches and spirits and water served all round. From remarks dropped it appeared the family were *en route* to Madame Tussauds. Well, they all look hale and hearty excepting the invalid, and they all vie in taking care of the invalid. He is helped first, has the dainty morsel, and is asked to take a second nip of the drinkable, whatever it may be. As, however, with cripples in general, his temperament is evidently excitable. The probability is that he does not sleep well o' nights. And it is more than likely the little sleep he gets will be murdered by this visit to the great waxwork exhibition. He will dream of the horrors he sees there, and we should not be surprised if this visit, planned in all kindness, and undertaken perhaps chiefly for the delectation of the invalid, does in reality retard his recovery. In the corner of the carriage is a lady to whom we have not yet given attention. Probably because the dress at once announces the avocation. She belongs to some sisterhood, but we hope not a nursing sisterhood, for the flapping white collar would certainly be in the way when administering to the sick. We have yet to notice another passenger, who, from the tool bag he carries, is doubtless a carpenter. He looks haggard, eager, thin, and ill, and it scarcely requires a hollow cough to inform us that the man is phthisical. We enter into conversation with him. He tells us he has been ailing for some time, suffering from "one cold a-top of another," and we are sorry to learn he is employed on an outside job this inclement weather. Many a workman succumbs from outdoor employment in the winter and spring whose lives would be preserved could they be employed on indoor work. Here is a new field for the philanthropist. Another type of third-class passenger we often meet with is the musical *artiste*, sometimes male sometimes female, known by the bag containing the instrument, which the artiste affects. Of course, there are artistes and artistes. At present we are concerned in regarding one only—evidently an infant prodigy, and her instrument the violin. Pallid faced, large eyed, sharp featured, it is clear that either study and practice or late hours, or both combined, are telling on this little girl's constitution. She will probably end in consumption or in premature loss of the musical talent which is now being abused. But our School Board education must be held responsible for not a little premature breaking down of children. Teaching children when they should be digesting food which they have not got, is not the way to secure a healthy, growing race. And—but we have arrived at our journey's end and must alight.

